# The effectiveness of interventions to improve uptake and retention of HIV-infected pregnant and breastfeeding women and their infants in prevention of mother-to-child transmission care programs in low- and middle-income countries: protocol for a systematic review and meta-analysis

**DOI:** 10.1186/s13643-015-0136-x

**Published:** 2015-11-03

**Authors:** Lisa M. Puchalski Ritchie, Monique van Lettow, Mina C. Hosseinipour, Nora E. Rosenberg, Sam Phiri, Megan Landes, Fabian Cataldo, Sharon E. Straus, For the PURE consortium

**Affiliations:** Department of Medicine, University of Toronto, Toronto, ON Canada; Li Ka Shing Knowledge Institute, St. Michaels Hospital, University of Toronto, Toronto, ON Canada; University Health Network, Toronto, ON Canada; Dignitas International, c/o PO Box 1071, Zomba, Malawi; Dalla Lana School of Public Health, University of Toronto, Toronto, Canada; Division of Infectious Diseases, University of North Carolina, Chapel Hill, NC USA; University of North Carolina Project, Lilongwe, Malawi; Lighthouse Trust, Lilongwe, Malawi; Department of Family and Community Medicine, University of Toronto, Toronto, ON Canada

**Keywords:** HIV, Prevention of mother-to-child transmission, Interventions, Retention, Uptake

## Abstract

**Background:**

Despite recent improvements, uptake and retention of mothers and infants in prevention of mother-to-child transmission (PMTCT) services remain well below target levels in many low- and middle-income countries (LMICs). Identification of effective interventions to support uptake and retention is the first step towards improvement. We aim to complete a systematic review and meta-analysis to evaluate the effectiveness of interventions at the patient, provider or health system level in improving uptake and retention of HIV-infected mothers and their infants in PMTCT services in LMICs.

**Methods/Design:**

We will include studies comparing usual care or no intervention to any type of intervention to improve uptake and retention of HIV-infected pregnant or breastfeeding women and their children from birth to 2 years of age attending PMTCT services in LMICs. We will include randomized controlled trials (RCTs), cluster RCTs, non-randomized controlled trials, and interrupted time series. The primary outcomes of interest are percentage of HIV-infected women receiving/initiated on anti-retroviral prophylaxis or treatment, percentage of infants receiving/initiated on anti-retroviral prophylaxis, and percentage of women and infants completing the anti-retroviral regimen/retained in PMTCT care. The following databases will be searched from inception: Ovid MEDLINE and EMBASE, The WHO Global Health Library, CAB abstracts, EBM Reviews, CINAHL, HealthSTAR and Web of Science databases, Scopus, PsychINFO, POPLINE, Sociological Abstracts, ERIC, AIDS Education Global Information System, NLM Gateway, LILACS, Google Scholar, British Library Catalogue, DARE, ProQuest Dissertation & Theses, the New York Academy of Grey Literature, Open Grey, The Cochrane Library, WHO International Clinical Trials Registry, Controlled Clinical Trials, and clinicaltrials.gov. Reference lists of included articles will be hand searched and study authors and content experts contacted to inquire about eligible unpublished or in progress studies. Screening, data abstraction, and risk of bias appraisal using the Cochrane Effective Practice and Organization of Care criteria will be conducted independently by two team members. Results will be synthesized narratively and a meta-analysis conducted using the DerSimonian Laird random effects method if appropriate based on assessment of clinical and statistical heterogeneity.

**Discussion:**

Our findings will be useful to PMTCT implementers, policy makers, and implementation researchers working in LMICs.

**Systematic review registration:**

PROSPERO CRD42015020829

**Electronic supplementary material:**

The online version of this article (doi:10.1186/s13643-015-0136-x) contains supplementary material, which is available to authorized users.

## Background

Although the incidence of pediatric HIV acquisition is falling, over 240,000 children were newly infected with HIV in 2013, primarily through mother-to-child transmission [1]. Prevention of mother-to-child transmission (PMTCT) therapeutic regimens have been proven to reduce the risk of mother-to-child transmission from 20–45 % to 2 % in non-breastfeeding populations and 5 % or less in breastfeeding populations [2]. However, despite recent improvements in PMTCT clinical service coverage in low- and middle-income countries (LMICs) from 10 % in 2004 to 67 % in 2013, uptake and retention of mothers and newborns in PMTCT clinical services remain well below target levels in many LMICs [1, 2]. PMTCT services begin with maternal HIV testing and counseling and for HIV-infected women include the following: initiation and maintenance of pregnant and nursing women and their infants on PMTCT medication regimens for the duration of treatment as defined by the specific regimen employed; and completion of appropriate infant HIV testing. As a result of the 2010–2015 PMTCT strategic vision, the World Health Organization (WHO) has called for renewed commitment and effort towards achieving universal PMTCT coverage. The identification of interventions to support PMTCT uptake and retention is the first step towards improvement.

To date, two systematic reviews have been published that specifically evaluated the effectiveness of interventions to improve PMTCT coverage. Both were limited to specific interventions—male involvement [3] and integration of services [4] —and found too few studies meeting inclusion criteria to assess or make recommendations regarding effectiveness. A third systematic review indentified nine completed studies and five ongoing trials which examined initiation of antiretroviral (ARV) treatment in pregnant women [5]. While the authors report several promising interventions for improving ARV initiation, the quality of evidence was insufficient to support recommendations. In addition, results for ARV initiation in pregnant women were not independently examined, and maternal retention in PMTCT care and exposed infant care were not assessed. However, in our preliminary search, we identified a number of additional interventions including integration of HIV and antenatal care, peer-based programs, and community health worker programs [6–8] that have been evaluated to improve PMTCT uptake and retention in LMICs.

Given the paucity of synthesized evidence to date, we propose to complete a systematic review to identify what interventions are effective in improving uptake and retention of HIV-infected mothers and their infants in PMTCT services in LMICs. While we anticipate a relatively small number of evaluations of any given intervention type, which may preclude meta-analysis, a narrative synthesis of the evidence to date is urgently needed to inform LMIC PMTCT program development and policy. With the exception of Option B+ (lifelong triple ARV therapy for all HIV+ pregnant and breastfeeding women, regardless of clinical stage or CD4 count) recommended by WHO in April 2012 for which evidence is not yet available, the effectiveness of PMTCT regimens is well established and will therefore not be included in the present search [9].

## Methods/Design

### Protocol

A preliminary systematic review protocol was developed based on the Cochrane Handbook [10]. The protocol was revised with input from the PURE Malawi Consortium, a research partnership of governmental, non-governmental, and academic organizations working to improve PMTCT programming in Malawi. The final protocol was registered with the PROSPERO database (CRD42015020829, available at: http://www.crd.york.ac.uk/PROSPERO/display_record.asp?ID=CRD42015020829#.VXHCNUZBn5I), with reporting of the protocol guided by the PRISMA-P [11].

### Eligibility criteria

We will include studies of HIV-infected pregnant and breastfeeding women and their children from birth to 2 years of age or termination of breastfeeding in LMICs. For the purpose of this review, we will utilize the EPOC filter to identify low- and middle-income countries [12] updated using the most recent World Bank World Country and Lending group classification [13] to define LMICs. Based on the unique challenges facing PMTCT health services in LMICs and intended use of the findings of this review to inform PMTCT service development in Malawi and other LMICs, we chose to limit the review to studies conducted in LMICs. Studies conducted only in high-income countries or where LMIC results cannot be separated will not be eligible for inclusion.

We will include studies comparing usual care or no intervention to any type of intervention (including patient, provider, or health system level interventions) to improve uptake and retention of HIV-infected pregnant or breastfeeding women and their children from birth to 2 years of age in PMTCT services. Patient level interventions are those focused on the patient and may include patient education programs, peer support programs, or efforts to improve patient support through engagement of partners or family members. Provider level interventions may include provider training, incentive programs, or tools to improve care provided. Health system level interventions may include restructuring of services and task shifting or other mechanisms to address human resource shortages.

The primary outcomes of interest are percentage of HIV-infected women receiving or initiated on ARV prophylaxis or treatment, percentage of infants born to HIV-infected mothers receiving or initiated on ARV prophylaxis, and percentage of women and infants retained in PMTCT care/completing the ARV regimen as defined by the PMTCT regimen utilized. Secondary outcomes of interest include the following: percentage of infants completing post-exposure HIV testing at 4–6 weeks after birth and percentage of infants completing post-exposure HIV testing at 6 weeks following termination of breastfeeding for all infants with known HIV exposure as recommend by the WHO [14]; percentage of HIV-exposed infants testing positive for HIV; and adverse events including negative impact(s) on resources/delivery and/or effectiveness of other health care programs (including economic impact), major (e.g., heart defects, neural tube defects, major limb malformations, hypospadias) or minor (e.g., syndactyly, cutis aplasia, accessory digit) congenital malformations, small for gestational age, premature delivery, still birth, and infant death within the first 2 years of life).

We will include controlled experimental studies (randomized controlled trials, cluster randomized controlled trials, non-randomized controlled trials) and controlled quasi-experimental studies (interrupted time series). We chose to include non-randomized controlled trials and quasi-experimental designs based on the results of our scoping searches, in which we found few randomized controlled trials that evaluated interventions to improve uptake and retention of HIV-infected women and their children in PMTCT services conducted in LMICs. Language of publication will be restricted to the language spoken by the study team and includes English only. No restrictions will be placed on publication status, study time frame, or duration of follow-up.

### Information sources and literature search

Our search strategy was developed in consultation with an experienced information specialist and peer reviewed by two additional information specialists with expertise in systematic reviews using the Peer Review of Electronic Search Strategies checklist [15].

We will search the following electronic databases from inception to June 2015 using medical subject headings (MeSH) and text words related to HIV, pregnancy, breastfeeding, mother-to-child transmission, interventions, treatment uptake and retention, and low- and middle-income countries, using MEDLINE (OVID interface, 1946 to July Week 4 onwards), EMBASE (OVID interface, 1974 onward), The WHO Global Health Library (http://www.globalhealthlibrary.net/php/index.php), CAB abstracts (OVID interface, 1973 onward), EBM Reviews (OVID interface, 1991 onward), CINAHL (EBSCOhost Research Databases interface, 19,814 onward), HealthSTAR (OVID interface, 1966 onward) and Web of Science databases (Thompson Reuters interface, 1975 onward), Scopus (Elsevier Interface, 1823 onward), PsychINFO (OVID interface, 1806 onward), POPLINE (www.POPLINE.org, 1970 onward), Sociological Abstracts (Proquest interface, 1953 onward), ERIC (EBSCOhost Research Databases interface, 1966 onward), AIDS Education Global Information System (http://www.aegis.org), NLM Gateway (http://gateway.nlm.nih.gov/), LILACS (http://bases.bireme.br/cgi-bin/wxislind.exe/iah/online/?IsisScript=iah/iah.xis&base=LILACS&lang=i), Google Scholar (https:*//*scholar*.*google*.*ca), British Library Catalogue (http://explore.bl.uk/primo_library/libweb/action/search.do?dscnt=1&dstmp=1445538063587&vid=BLVU1&fromLogin=true), DARE (LexisNexis Academic interface, 2010 onward), ProQuest Dissertation & Theses (Proquest Interface, 1637 onward), the New York Academy of Grey Literature (http://library.tmc.edu/website/new-york-academy-of-medicine-library-grey-literature-collection/), OpenGrey (http://www.opengrey.eu/), The Cochrane Library (http://www.cochranelibrary.com/), WHO International Clinical Trials Registry (http://www.who.int/ictrp/en/), Controlled Clinical Trials (http://www.controlled-trials.com/), and clinicaltrials.gov (https://clinicaltrials.gov/). In addition, we will search reference lists of included articles and will contact experts in the field to inquire about eligible unpublished or in progress studies. Low- and middle-income countries will be searched utilizing the EPOC LMIC filter [12], updated based on the most recent World Bank LMIC list [13], see Additional file 1 for full MEDLINE search strategy. We will employ the Cochrane highly sensitive search strategy for identifying randomized trials in OVID MEDLINE: sensitivity and precision maximizing version [16], with the following two changes: Random* was used in place of randomized or randomly and trials ti was not used as an isolated term.

### Study selection process

All titles and abstracts identified by the database search will be entered into a reference manager and duplicates removed manually into the duplicate folder, with companion papers for the same study retained for further evaluation at the full article phase of the review. Citations will be screened in two phases, level 1 (titles and abstracts) and level 2 (full-text review). A screening checklist will be developed and pilot tested by the reviewers on a random sample of 50 citations for each screening phase. Inter-rater agreement will be calculated for the pilot test and the form revised and re-piloted if percent agreement is <90 %. Once adequate agreement has been achieved, two team members will independently screen citations using the screening checklist. Differences at each stage will be resolved by consensus and if necessary through discussion with a third team member who is a content expert. Reference lists of included studies will be reviewed independently by the same two team members and again differences resolved through consensus and if necessary consultation with a third team member. A review log will be maintained in order to provide a record of resolution of discrepancies, decisions regarding studies described in >1 report, and reasons for exclusion.

### Data abstraction and management

Data abstraction forms will be developed and pilot tested. Two team members will independently abstract directly into excel spreadsheets, corresponding to outcome tables, with additional space for comments and reasons for exclusion. Inter-rater reliability will be measured for data abstraction on a sample of excluded and included articles (approximately 10 %), and if percent agreement is found to be below 90 %, abstraction is conducted by a third team member. All discrepancies will be reviewed and consensus reached through discussion.

Data abstraction will be based on the PICOST [17] format including population, intervention, comparator, context, outcomes, study DESIGN, and time frame. Population characteristics to be abstracted include maternal age, number of children, marital status, place of residence (rural/urban), level of education, primary language, first infant HIV testing (4–6 weeks), and at end of study. Study characteristics of interest include study design, country and geographical location within country (rural/urban), setting (home, hospital or health center clinic, maternity ward), detailed description of intervention and comparator (usual care/no intervention), number of participants per group at study baseline and follow-up, duration of intervention and follow-up period, source of data (self-report, clinical records, pill counting), and publication status. Outcome data to be abstracted include percentage of HIV-infected women and their infants receiving or initiating PMTCT treatment, retained in or completing PMTCT as defined by the PMTCT regimen(s) used. Where data necessary for analysis are missing, corresponding authors will be contacted.

Although improved in recent years, examples of cluster trails inappropriately analyzed (without adjustment for cluster randomization) may be found among older trials. Data on appropriateness of analysis will be abstracted and reported as part of the review findings.

### Methodological quality/risk of bias appraisal

Risk of bias assessment will be conducted using the Cochrane Effective Practice and Organization of Care (EPOC) criteria for assessing risk of bias [18]. Categories of bias assessed by this tool for randomized controlled trials, and non-randomized controlled trails include: allocation concealment, measurement of baseline characteristics and outcomes, management of incomplete data, blinding of outcome assessment, protection against contamination, selective reporting, and other categories of bias [18]. Categories of bias assessed by this tool for interrupted time series and repeated measures studies include independence of intervention from other changes, pre-specification of the intervention effect shape, effect of data collection on the intervention, allocation concealment, management of incomplete data, selective reporting, and other sources of bias [18]. Two team members will independently assess the studies for risk of bias at both study and outcome levels with disagreement resolved by consensus and discussion with a third team member if necessary. Studies will not be excluded based on risk of bias assessment, but the information will be used in the analysis and reporting of findings. Risk of bias will be categorized as low, high, or unclear risk of bias, using the EPOC-suggested risk of bias criteria [18]. We have elected not to use GRADE for this review given that the review findings are urgently needed to inform PMCTC program development and policy and that the need to build capacity in the use of grade across the team which would significantly prolong the review timeline.

Risk of publication bias will be examined using funnel plots. For studies in which selected reporting bias is suspected, planned outcomes will be reviewed for registered trials and authors contacted for missing outcomes and for unregistered trials, and risk of selected reporting bias rated as unclear if response not received within 8 weeks of our initial email request.

### Evidence synthesis

A flow diagram will be utilized to visually present the results of the search strategy and reasons for exclusion of articles. Included articles will be synthesized and reported narratively and in tabular form to provide an overview of findings, assessment of bias and its potential impact on reported findings, and strengths and weaknesses of included studies. Summary statistics for continuous outcomes will be expressed as mean difference and standardized mean difference with 95 % CIs, for outcomes reported using the same and different scales, respectively. Summary statistics for dichotomous data will be expressed as risk ratio with 95 % CI.

If meta-analysis is possible, it will be conducted using the DerSimonian Laird random effects method. Summary statistics will be expressed as risk ratios with 95 % confidence interval. Clinical heterogeneity will be determined based on patient, intervention, and outcome characteristics of included studies. Statistical heterogeneity will be determined visually and the impact of heterogeneity assessed using the I^2^ test, with I^2^ of 75 % considered significant. Given the time constraints for this review, re-analysis for unit of analysis errors will not be conducted and cluster trials with unit of analysis errors will be excluded from the primary meta-analysis, and their impact assessed with sensitivity analysis comparing meta-analysis with and without studies with unit of analysis errors included. Interventions at the patient, provider, and health system level will be reported separately and analyzed separately if possible to do so.

## Discussion

The findings of this review will have significant implications for PMTCT program development and policy in LMICs. If high-quality evidence of intervention effectiveness is identified, this will provide important guidance to ongoing efforts to address low rates of uptake and retention of HIV-infected mothers and their infants in PMTCT services in LMICs. If high-quality evidence is not identified, findings of the systematic review may identify gaps in evidence and promising interventions providing direction for future intervention research.

To ensure our findings reach audiences who may benefit from the review findings, we plan to disseminate the results through publication in open access peer-reviewed journals, presentations at relevant international conferences, and direct communication within the professional networks of PURE consortium members.

## Additional file

Additional file 1:
**Ovid MEDLINE search strategy.** (DOC 39 kb)

## Abbreviations

ARV: anti-retroviral
EPOC: Effective Practice and Organization of Care
HIV: human immunodeficiency virus
LMIC: low- and middle-income country
MeSH: medical subject headings
PMTCT: prevention of mother-to-child transmission
WHO: World Health Organization
