# Supplementary material for: The effectiveness of interventions to improve uptake and retention of HIV-infected pregnant and breastfeeding women and their infants in prevention of mother-to-child transmission care programs in low- and middle-income countries: protocol for a systematic review and meta-analysis
Source: Syst Rev. 2015 Nov 3;4:144. doi: 10.1186/s13643-015-0136-x (PMC4630855; doi:10.1186/s13643-015-0136-x)
Supplement: Additional file 1: — Ovid MEDLINE search strategy. (DOC 39 kb) [file 13643_2015_136_MOESM1_ESM.doc]

Additional File 1: Search Strategy Ovid MEDLINE(R) <1946 to June Week 1 2015>:

--------------------------------------------------------------------------------

Pregnant / Breastfeeding Women

1 Pregnant Women/ (5226)

2 exp Breast Feeding/ (26666)

3 Milk, Human/ (15697)

4 Infectious Disease Transmission, Vertical/ (12256)

5 fetus/ (68631)

6 exp pregnancy/ (723003)

7 peripartum period/ (427)

8 exp Postpartum Period/ (49233)

9 exp pregnancy complications/ (345863)

10 exp Maternal Health Services/ (35913)

11 pregnan*.mp,kw,kf. (778553)

12 gestat*.tw,kw,kf. (144054)

13 breastfeed*.mp,kw,kf. (13469)

14 (breast adj2 feed*).mp,kw,kf. (30938)

15 (breast adj2 milk).mp,kw,kf. (8972)

16 breastmilk.tw,kw,kf. (683)

17 human milk.tw,kw,kf. (7840)

18 lactat*.mp,kw,kf. (165010)

19 (milk adj2 eject*).tw,kw,kf. (704)

20 (milk adj2 let*-down).tw,kw,kf. (68)

21 ((expectant or expecting) adj2 wom#n).mp,kw,kf. (182)

22 parturit*.tw,kw,kf. (11506)

23 birth*.mp,kw,kf. (259925)

24 childbirth*.mp,kw,kf. (14074)

25 child-birth*.mp,kw,kf. (491)

26 deliver*.mp,kw,kf. (474171)

27 puerper*.mp,kw,kf. (21074)

28 breastfed.tw,kw,kf. (3524)

29 mtct.tw,kw,kf. (559)

30 pmtct.tw,kw,kf. (725)

31 (vertical adj2 transmission*).tw,kw,kf. (4511)

32 f?etus*.mp,kw,kf. (137278)

33 f?etal.mp,kw,kf. (302029)

34 (breast adj2 fed*).tw,kw,kf. (5276)

35 in-utero.tw,kw,kf. (20490)

36 (intrauterine or intra-uterine).tw,kw,kf. (42420)

37 (trans-placent* or transplacent*).tw,kw,kf. (5212)

38 (f?eto-maternal or f?etomaternal).tw,kw,kf. (2682)

39 (parent* adj2 (child* or infant* or baby or babies or neonat* or newborn*)).tw,kw,kf. (28605)

40 mother*.tw,kw,kf. (147803)

41 (nursing adj2 (infant* or baby or babies or neonat* or newborn*)).tw,kw,kf. (1319)

42 (prenatal* or pre-natal*).tw,kw,kf. (70920)

43 (perinatal* or peri-natal*).tw,kw,kf. (51747)

44 (post-natal* or postnatal*).tw,kw,kf. (85370)

45 (antenatal* or antenatal*).tw,kw,kf. (23135)

46 (antepartum* or ante-partum*).tw,kw,kf. (4566)

47 (postpartum* or post-partum*).tw,kw,kf. (40829)

48 maternal*.tw,kw,kf. (172644)

49 or/1-48 (1763167)

HIV/AIDS

50 exp HIV Infections/ (233689)

51 exp HIV/ (83825)

52 HIV Long-Term Survivors/ (607)

53 AIDS Serodiagnosis/ (6107)

54 hiv.mp,kw,kf. (263320)

55 Human T-Cell Leukemia Virus.mp,kw,kf. (2850)

56 htlv-iii.mp,kw,kf. (1652)

57 (acquired adj2 immun* adj2 (syndrome* or virus*)).mp,kw,kf. (86030)

58 (human* adj2 immun* adj2 deficien* adj2 virus*).mp,kw,kf. (491)

59 (human* adj2 immun* adj2 virus*).mp,kw,kf. (76929)

60 (syndrome* adj2 lymphadenopath*).tw,kw,kf. (335)

61 slim disease.tw,kw,kf. (25)

62 lymphadenopathy-associated virus*.mp,kw,kf. (295)

63 lav-htlv-iii.mp,kw,kf. (211)

64 sbl-6669.mp,kw,kf. (16)

65 lav-2.mp,kw,kf. (25)

66 (acquired adj2 immun* adj2 deficien* adj2 syndrome*).tw,kw,kf. (5057)

67 (aids adj10 (disease* or syndrome*)).mp,kw,kf. (27876)

68 (aids adj1 related).tw,kw,kf. (6614)

69 htlv*.tw,kw,kf. (11427)

70 hiv##.mp,kw,kf. (1760)

71 or/50-70 (325026)

Patient uptake / dropouts / participation

72 Patient Dropouts/ (6786)

73 exp "Patient Acceptance of Health Care"/ [includes treatment refusal MeSH] (171083)

74 exp Consumer Participation/ (32566)

75 dropout*.tw,kw,kf. (6483)

76 (uptake or up-take).tw,kw,kf. (248330)

77 (drop* adj1 out$1).tw,kw,kf. (8228)

78 (refusal* or refuse$1 or refusing).tw,kw,kf. (23366)

79 (patient* adj2 (elope or elope$1 or eloping)).tw,kw,kf. (4)

80 (non complian* or noncomplian*).tw,kw,kf. (9990)

81 complian*.tw,kw,kf. (84306)

82 (uncooperat* or unco-operat* or un-co-operat*).tw,kw,kf. (1028)

83 (cooperat* or co-operat*).tw,kw,kf. (102475)

84 (non-accept* or nonaccept*).tw,kw,kf. (592)

85 accept*.tw,kw,kf. (279089)

86 (nonparticipat* or non-participat*).tw,kw,kf. (1298)

87 participat*.tw,kw,kf. (322007)

88 (nonadher* or non-adher*).tw,kw,kf. (10638)

89 adher*.tw,kw,kf. (114637)

90 (retain* or retention*).tw,kw,kf. (244370)

91 (non-attend* or nonattend*).tw,kw,kf. (1453)

92 attend*.tw,kw,kf. (110407)

93 (comply* or complies or complian*).tw,kw,kf. (91550)

94 (non-comply* or noncomply* or non-complian* or noncomplian*).tw,kw,kf. (10004)

95 reluctan*.tw,kw,kf. (8504)

96 ((healthcare or care or advice or medical or information) adj3 seek$3).tw,kw,kf. (15252)

97 (disengag* or dis-engag*).tw,kw,kf. (2812)

98 engag*.tw,kw,kf. (82419)

99 avoid*.tw,kw,kf. (237366)

100 ut.fs. (144195)

101 ignor*.tw,kw,kf. (27215)

102 reject*.tw,kw,kf. (82472)

103 (non-embrac* or nonembrac*).tw,kw,kf. (0)

104 (un-embrac* or unembrac*).tw,kw,kf. (1)

105 (embrace* or embracing).tw,kw,kf. (7691)

106 (un-accept* or unaccept*).tw,kw,kf. (14546)

107 (unadher* or un-adher*).tw,kw,kf. (14)

108 no-show*.tw,kw,kf. (484)

109 (follow* adj1 up).tw,kw,kf. (638770)

110 incent*.tw,kw,kf. (17823)

111 enabl*.tw,kw,kf. (214935)

112 disincent*.tw,kw,kf. (859)

113 utiliz*.tw,kw,kf. (319558)

114 (inclin* or disinclin*).tw,kw,kf. (12034)

115 or/72-114 (2984236)

Study type / characteristics

116 randomized controlled trial.pt. (387105)

117 exp Randomized controlled trial/ (387132)

118 exp Randomized Controlled Trials as Topic/ (97414)

119 clinical trial.pt. (490674)

120 Double-Blind Method/ (128228)

121 Placebos/ (32662)

122 clinical trials as topic/ (171490)

123 evaluation research/ (119973)

124 program evaluation/ (47548)

125 Feasibility Studies/ (45412)

126 Pilot Projects/ (85700)

127 Evaluation Studies as Topic/ (119973)

128 Cost-Benefit Analysis/ (61646)

129 (random* or non-random* or unrandom* or nonrandom*).mp,kw,kf. (874470)

130 placebo*.mp,kw,kf. (168179)

131 rct*1.tw,kw,kf. (17367)

132 ((singl* or doubl* or trebl* or tripl*) adj1 (mask* or blind* or dumm*)).mp,kw,kf. (176744)

133 evaluat*.mp,kw,kf. (2416275)

134 effectiv*.mp,kw,kf. (1149619)

135 sustainab*.mp,kw,kf. (23041)

136 feasib*.mp,kw,kf. (177882)

137 appropriateness.mp,kw,kf. (12458)

138 efficac*.mp,kw,kf. (507876)

139 impact*.mp,kw,kf. (537916)

140 (pilot adj2 (project* or study or studies)).mp,kw,kf. (103303)

141 cost-effectiv*.mp,kw,kf. (73309)

142 (cost*1 adj2 benefit*1).mp,kw,kf. (69472)

143 (interrupt* adj2 time).mp,kw,kf. (1224)

144 or/116-143 (4705604)

Lower middle income countries

145 Developing Countries/ (63034)

146 (lmic or lmics or lami countr*).mp,sh,kf,in,jn,nj,ia,cp,pb. (534)

147 ((developing or less* developed or under developed or underdeveloped or middle income or low* income or underserved or under served or deprived or poor*) adj (countr* or nation? or population? or world)).hw,kf,ti,ab,cp,in,jn,nj,ia,cp,pb,mp. (106086)

148 (Afghan* or Albania* or Algeria* or Angola* or Antigua* or Barbud* or Argentin* or Armenia* or Aruba* or Azerbaijan* or Bahrain* or Bangladesh* or Barbad* or Benin* or Byelarus* or Byelorus* or Belarus* or Belorus* or Beliz* or Bhutan* or Bolivia* or Bosnia* or Herzegovin* or Hercegovin* or Botswan* or Brasil* or Brazil* or Bulgaria* or Burkina Faso* or Burkina Fasso* or Upper Volta* or Burundi* or Urundi* or Cambodia* or Khmer Republic or Kampuchea* or Cameroon* or Cameron* or Cape Verde* or Central African Republic or Chad* or Chile* or China or chinese or Colombia* or Comoros* or Comoro Islands or Comores or Mayott* or Congo* or Zair* or Costa Rica* or Cote d'Ivoire or Ivory Coast or Croatia* or Cuba* or Cyprus or cyprian or Czechoslovakia* or Czech Republic or Slovakia* or Slovak Republic or Djibouti* or French Somaliland or Dominica* or East Timor or East Timur or Timor Leste or Ecuador* or Egypt* or United Arab Republic or El Salvador* or Eritrea* or Estonia* or Ethiopia* or Fiji* or Gabon* or Gambia* or Gaza* or Georgia Republic or Georgian Republic or georgian or Ghana* or Gold Coast or Greece or greek or Grenada* or Guatemala* or Guinea* or Guam* or Guiana* or Guyana* or Haiti* or Hondura* or Hungar* or India* or Maldiv* or Indonesia* or Iran* or Iraq* or Isle of Man or Jamaica* or Jordan* or Kazakh* or Kenya* or Kiribati* or Korea* or Kosovo* or Kyrgyz* or Kirghiz* or Kirgiz* or Lao PDR or Laos* or Latvia* or Leban* or Lesotho* or Basutoland or Liberia* or Libya* or Lithuania* or Macedonia* or Madagascar* or Malagasy Republic or Malay* or Sabah* or Sarawak* or Malawi* or Nyasaland* or Mali* or Malta* or Marshall Island* or Maurit* or Agalega Island* or Mexic* or Micronesia* or Middle East* or Moldova* or Moldovia* or Mongolia* or Montenegr* or Morocc* or Ifni* or Mozambiq* or Myanmar* or Myanma or Burma* or Namibia* or Nepal* or Netherlands Antill* or New Caledonia* or Nicaragua* or Niger* or Northern Mariana Island* or Oman* or Muscat* or Pakistan* or Palau* or Palestin* or Panama* or Paragua* or Peru* or Phi?lippin* or Poland or polish or Portug* or Puerto Ric* or Romania* or Rumania* or Roumania* or Russia* or Rwanda* or Ruanda* or Saint Kitts* or St Kitts or Nevis* or Saint Lucia* or St Lucia* or Saint Vincent* or St Vincent* or Grenadin* or Samoa* or Navigator Island* or Sao Tome* or Saudi Arabia* or saudi or Senegal* or Serbia* or Montenegr* or Seychelles or Sierra Leone or Slovenia* or Sri Lanka* or Ceylon* or Solomon Islands or Somalia* or South Africa* or Sudan* or Surinam* or Swaziland or swazi or Syria* or Tajik* or Tadjik* or Tadzhik* or Tanzania* or Thailand or thai or Togo or Togolese Republic or Tonga* or Trinidad* or Tobag* or Tunisia* or Turkey or turkish or Turkmenistan* or Turkmen* or Uganda* or Ukrain* or Urugua* or USSR or Soviet Union or Union of Soviet Socialist Republics or Uzbek* or Vanuat* or New Hebrides or Venezuela* or Vietnam* or Viet Nam* or West Bank or Yemen* or Yugoslavia* or Zambia* or Zimbabw* or Rhodesia* or cabo verd*).hw,kf,ti,ab,cp,in,jn,nj,ia,cp,pb,mp. (4641336)

149 or/145-148 (4677916)

Full topic

150 49 and 71 and 115 and 144 and 149 (3309)

151 exp animals/ not (exp animals/ and exp humans/) (4003250)

Full topic minus animal-only studies

152 150 not 151 (3291)

***************************
